# D-Limonene Promotes Anti-Obesity in 3T3-L1 Adipocytes and High-Calorie Diet-Induced Obese Rats by Activating the AMPK Signaling Pathway

**DOI:** 10.3390/nu15020267

**Published:** 2023-01-04

**Authors:** Jin-Ting Liao, Yu-Wen Huang, Chih-Yao Hou, Jyh-Jye Wang, Chih-Chung Wu, Shu-Ling Hsieh

**Affiliations:** 1 Department of Seafood Science, National Kaohsiung University of Science and Technology, Kaohsiung 81157, Taiwan; 2 Department of Nutrition and Health Science, Fooyin University, Kaohsiung 83102, Taiwan; 3 Department of Food and Nutrition, Providence University, Taichung 43301, Taiwan

**Keywords:** 3T3-L1 adipocyte, AMPK signaling pathway, anti-obesity, limonene, high-calorie diet-induced obese rat

## Abstract

D-limonene (LIM) is a common monoterpene compound, principally found in citrus essential oils. This study investigated the anti-obesity effect of LIM on the 5′-adenosine monophosphate (AMP)-activated protein kinase (AMPK) signaling pathway in 3T3-L1 adipocytes and high-calorie diet-induced obese rats and confirmed the optimally effective dose of LIM. The 3T3-L1 adipocytes were treated with 0.05–0.4 mg/mL LIM for 10 days and oil red O and triglyceride (TG) content were used to determine the levels of lipid accumulation. The results showed that more than 0.05 mg/mL LIM inhibited lipid accumulation by reducing oil red O in 3T3-L1 adipocytes. Masses of 0.2 and 0.4 mg/mL LIM also decreased the TG contents in 3T3-L1 adipocytes. On the other hand, Wistar rats were given high-calorie diets, combined with LLIM (154 mg/kg) and HLIM (1000 mg/kg) treatments, for 16 weeks. The result shows that LLIM and HLIM decreased body weight, total fat tissue weight, and serum low-density lipoprotein-cholesterol (LDLc) levels. HLIM reduced serum TG and increased serum lipase and high-density lipoprotein-cholesterol (HDLc) levels. Moreover, the anti-obesity metabolic pathway showed that LIM (>0.05 mg/mL) in 3T3-L1 adipocytes and LIM (>154 mg/kg) in high-calorie diet-induced obese rats could activate the AMPK signaling pathway. The activated AMPK regulated the mRNA expression related to adipogenesis (PPARγ, C/EBPα, FABP4), lipogenesis (SREBP-1c, ACC, FAS), and lipolysis (ATGL, HSL) to inhibit obesity. This finding demonstrates that LIM has anti-obesity properties. Namely, it is seen that LIM acts by regulating the AMPK signaling pathway in 3T3-L1 adipocytes and high-calorie diet-induced obese rats. In terms of dose–response, LIM (154 mg/kg) would be an optimal effective dose for anti-obesity induced by a high-calorie diet.

## 1. Introduction

Obesity has recently become a significant clinical problem globally. A state of excessive body fat accumulation, caused by an imbalance between energy intake and energy expenditure, is known as obesity [1]. As of 2020, there are obese people about 760 million in the world’s population, and it is projected that there will 1 billion obese adults worldwide in 2030 [2]. It is noteworthy that obesity is related to the development of metabolic disorders, including hypertension, type 2 diabetes, hyperlipidemia, and cardiovascular disease [3]. White adipose tissue (WAT) has complex metabolic functions. When energy uptake surpasses energy expenditure, excessive energy is synthesized into triglyceride (TG) and stored in WAT [4]. WAT becomes extremely dysfunctional and fails to store excessive TG during obesity. Additionally, excessive TG is deposited into the liver tissue [5]. Finally, this causes dyslipidemia involved in abnormal liver lipid metabolism, including increased TG, free fatty acid (FFA), low-density lipoprotein cholesterol (LDLc), and decreased high-density lipoprotein cholesterol (HDLc) [6]. Therefore, the regulation of WAT via decreasing adipogenesis, lipogenesis, and promoting lipolysis can be a potential therapeutic route in the treatment of obesity and related metabolic dysfunction.

5′-adenosine monophosphate (AMP)-activated protein kinase (AMPK) has been investigated as a critical regulator of energy balance in WAT [7]. AMPK is a sensor in cellular homeostasis that regulates the anabolic and catabolic mechanisms of energy balance [7]. Activation of AMPK by phosphorylation inhibits adipogenesis and lipogenesis, including the inactivation of peroxisome proliferator-activated receptor γ (PPARγ), CCAAT/enhancer binding proteins α (C/EBPα), acetyl-CoA carboxylase (ACC), fatty acid synthesis (FAS), and sterol regulatory element-binding protein 1c (SREBP-1c) [7,8,9]. Activation of AMPK also promotes the activity of hormone-sensitive lipase (HSL) and adipose triglyceride lipase (ATGL), both of which are factors related to lipolysis [7,8,9]. A previous study confirmed that calebin-A (a terpenoid) improved total fat mass accumulation and dyslipidemia in high-fat diet-induced rats by regulating the AMPK signaling pathway. This included the protein expression of PPARγ, C/EBPα, FAS, and ACC [10]. Sri Devi, Ashokkumar and Citral (2018) reported that citrus inhibited lipid accumulation via the reduced protein expression of PPARγ, C/EBPα, FAS, and SREBP-1c in 3T3-L1 adipocytes [11]. Consequently, activating AMPK activity may be a workable treatment approach for obesity.

Many studies have evidenced that dietary phytochemicals may be used as anti-obesity agents because they inhibit the differentiation of adipocytes and stimulate lipolysis [12]. D-limonene (l-methyl-4-(1-methylethenyl)-cyclohexane, LIM) is a volatile monoterpene compound produced by plants [13]. LIM is principally found in citrus essential oils, such as lemon, orange, and grapefruit oils [13]. LIM also exists in citrus pulp, which is the main source of human D-limonene intake [14]. It was reported that LIM had low toxicity and showed no evidence of risk in animal studies [15]. Therefore, LIM has been used to evaluate many biological activities, such as anti-oxidation, anti-inflammation, and the ability to regulate blood pressure [16,17,18]. Previous studies have shown that LIM has anti-obesity properties in 3T3-L1 adipocytes and high-fat diet-induced obese rats [19,20]. However, few studies have thoroughly reported the effect of LIM on anti-obesity-related metabolism and its optimal effective dose. Thus, this study evaluated the optimal dose of LIM on anti-obesity and the effect of its lipid metabolism on the AMPK signaling pathway.

## 2. Materials and Methods

### 2.1. Chemicals

D-limonene, sodium bicarbonate (NaHCO₃), 3-isobutyl-methylxanthine (IBMX), dexamethasone, insulin, oil red O, 3-(4,5-dimethyazol-2-yl)-2,5-diphenyltetrazolium bromide (MTT), formaldehyde, triton X-100, TRIzol reagent, and trichloromethane were supplied from Sigma-Aldrich (St. Louis, MI, USA). GC-grade acetone, isopropanol, and methanol were purchased by J.T. Baker brand chemistries (Phillipsburg, NJ, USA). Penicillin/streptomycin (P/S), fetal bovine serum (FBS), trypsin, and Dulbecco’s modified eagle high-glucose medium (DMEM) were obtained from Gibco (New York, NY, USA). Phosphate-buffered saline (PBS) was acquired from Uni-onward (Taipei, Taiwan). RNase, M-MLV reverse transcriptase, and oligo dT were used with commercially available kits from Promega (Madison, WI, USA). Primer and 2×SYBR Frist MM were obtained from Topgen (Kaohsiung, Taiwan), according to the manufacturer’s instructions. All the other reagents and chemicals were purchased from commercial companies.

### 2.2. Cell Culture and Adipocyte Differentiation

3T3-L1 adipocytes were differentiated from 3T3-L1 pre-adipocytes. 3T3-L1 pre-adipocytes were differentiated as previously described [14]. 3T3-L1 pre-adipocytes were seeded in a 3 cm dish at a density of 5 × 10^4^ cells per dish for 24 h (0 days). After confluence in 2 days, the DMEM medium was removed (containing 10% FBS). 3T3-L1 pre-adipocytes were cultured in the differentiation medium (MDI) (containing DMEM supplemented with IBMX (0.5 mM), dexamethasone (1 μM), and insulin (10 μg/mL)) for 2 days. Starting from the 4th day, cells were cultured in the adipocyte maintenance medium containing DMEM supplemented with 10 μg/mL insulin, and the medium was changed every day until the 8th day. The cells were grown in DMEM for two days. 3T3-L1 adipocyte differentiation was completed after 10 days.

### 2.3. Cell Viability Assay

The MTT assay, used to measure cell viability, was described previously [14]. The 3T3-L1 pre-adipocytes were seeded into 3 cm dishes at a density of 5 × 10^4^ cells and were incubated for 24 h. After incubation, the cells were treated with different concentrations (0.05, 0.1, 0.2, and 0.4 mg/mL, dissolved in DMSO) of LIM and differentiation for 10 days. All samples were treated with the above concentrations and differentiated in the cell model of this study. The 3T3-L1 adipocytes differentiation method is shown under Section 2.2. After removing the supernatant, 1 mL of MTT solution (0.1 mg/mL) was added to each dish and the viable cells were labelled. The dishes were incubated in a humid incubator for three hours. The optical density (OD) at 570 nm absorbance was utilized to determine the cell viability, after dissolving the formazan in 1 mL of isopropanol. The viability of untreated cells (control) was set at 100%, to calculate the percentage of viable cells in other groups.

### 2.4. Intracellular Lipid Assay

#### 2.4.1. Oil Red O Staining Assay

The cells were gently washed twice in 1 mL PBS and fixed in 10% formaldehyde for 5 min at room temperature. The cells were stained for 30 min using 1 mL of the oil red O solution. After 30 min, the oil red O solution was removed and washed twice in water. The stain images were taken with a microscope (Olympus, Tokyo, Japan). Then, cellular lipid droplet levels were measured by dissolving oil-red O stain with 1 mL isopropanol and using a spectrophotometer at OD 510 nm. The oil-red content of the untreated cells (control) was set at 100%, to calculate the percentage of oil-red levels in other groups.

#### 2.4.2. Intracellular Triglyceride Assay

A previously described procedure was used to determine the intracellular TG levels with only minor modifications [14]. A volume of 1 mL of PBS was used to wash the cells, twice. Then, 70 μL of PI buffer was added (containing 0.5 mM PMSF, 8 mM KH_2_PO_4_, 12 mM K_2_HPO_4_, 0.5% Triton X-100, and 1.5% KCl) and scraped into a 1.7 mL tube, and the solution was homogenized by sonication (Qsonica, CT, USA) for 30 s (80% amplitude, 20 kHz frequency, and 125 watts power rating). The total TG in the lysate was determined by TG assay kits (TR212; RANDOX, Ireland, UK) and protein concentration was measured by the Lowry method [21]. The formula of data was calculated using the following equation: TG deposition level = TG (mg)/protein (mg).

### 2.5. Animals and Diet

Male Wistar rats (aged 6 weeks; 250 ± 20 g) were obtained from BioLASCO Taiwan Co., Ltd. in Taipei. The animal study protocols were accepted by the Animal Care and Use Committees (IACUC) at the National Kaohsiung University of Science and Technology (Kaohsiung, Taiwan) (IACUC number: 0109-AAAP-014). The rats stayed under 22 ± 2 °C on 12 h light/dark cycle for one week to adapt. After the adaptation period, Wistar rats were weighed and randomly separated into four groups (six rats/group): normal diet as a control group (ND, 13.5% of energy as fat, Laboratory Rodent diet 5001, Lab5001; New Taipei City, Taiwan), high-calorie diet (HD, 45% of energy as fat), HD supplemented with low-dose LIM (HD + LLIM, 154 mg/kg) and HD supplemented with high-dose LIM (HD + HLIM, 1000 mg/kg) for 16 weeks. The LLIM dose was selected from this cell model study by calculating the blood volume and body weight of rats, and the HLIM dose referred to the dose in previous reports by converting the food intake of the rats [18]. A high-calorie diet was created in soybean oil and sugar and mixed with Lab5001. LIM was given four times a week via oral gavage after being dissolved in soybean oil. The ND and HD groups also consumed soybean oil by oral gavage.

### 2.6. Animal Growth Characteristics Assay

During the experimental procedures, body weight and food intake were assessed twice a week. Weight gain, Lee’s index, organ weight, feed conversion rate (FCR), and total fat tissue weight were used in the following equation: Weight gain (g) = (final weight (g)-initial weight (g))/final weight (g) *100, Lee’s index = (∛(body) weight(g)/body length (cm) [22,23], organ weight (%) = (organ weight (g)/body weight (g))*100, FCR (%) = weight gain (g)/total amount of food intake (g), and total fat tissue weight = epididymal adipose tissue (g) + retroperitoneal adipose tissue (g) + mesenteric adipose tissue (g). At the end of the study, rats starved for 24 h were sacrificed by carbon dioxide (CO_2_) asphyxiation and blood samples were collected from the heart. The liver, spleen, kidney, and fat tissues (epididymal, mesenteric, and retroperitoneal fat tissues) were immediately removed, weighed, and photographed. A portion of the epididymal adipose tissue was removed and preserved in formaldehyde. After it was determined that the blood samples could clot at room temperature for 4 h, the blood samples were centrifuged for 15 min at 2438× *g* and 4 °C. The upper fraction (serum) was drawn and stored at −20 °C until further assay.

### 2.7. Biochemical Parameters Assay

Commercial detection kits for TG (TR212), LDLc (CH2657), HDLc (CH2655), total cholesterol (TC; CH202), FFA (ab65341), lipase (LI118), and ketone body (RB1007) were obtained from RANDOX (Ireland, UK). Kits for detecting alanine aminotransferase (ALT; ab105134), aspartate aminotransferase (AST; ab105135), creatinine (ab65340), potassium (K, ab252904), and sodium (Na; ab211096) were obtained from Abcam (Cambridge, UK). Serum biochemical parameters were analyzed via an enzymatic colorimetric method, using commercial assay kits according to the manufacturer’s instructions.

### 2.8. Histological Assay

The epididymal adipose tissue was extracted and fixed in 10% formaldehyde for 24 h. The tissues were dehydrated with ethanol, embedded in paraffin wax, and sectioned into 8 μm thick slices. The slices were stained with haematoxylin and eosin (H&E) [24]. After sealing the slide with neutral gum, the morphological alterations of adipocytes were seen using an optical microscope (Olympus, Tokyo, Japan), and the cross-sectional areas of adipocytes were computed using Image-J software (U. S. National Institutes of Health, Bethesda, MD, USA).

### 2.9. mRNA Expression Assay

The mRNA expression was measured through real-time PCR (qPCR). Total RNA was isolated from 3T3-L1 adipocytes and epididymal adipose tissue by using the TRIzol reagent. Briefly, the cells or tissues were homogenized in 1 mL of TRIzol reagent. After centrifugation, RNA was extracted with trichloromethane and precipitated with isopropyl alcohol, then resuspended in 30–100 μL of DEPC-treated water. Then, total RNA was assessed in Epoch microvolume spectrophotometers (Bio-Tek, VT, USA) for DNA quantification. We used d-NTP, oligo-dT, M-MLV reverse, and RNase transcriptase to perform first-strand complementary DNA synthesis. Subsequently, the gene expression levels were verified using the qPCR (LightCycler^®^ 96 qPCR System, Roche Life Science, Basel, Switzerland). Amplifications were carried out using 5 μL of SYBR green, 2 μL of cDNA, 0.5 μL of each primer, and 2.5 μL of ddH_2_O under the following PCR regime: initial denaturation at 95 °C for 120 s (carried out for 1 cycle); 40 cycles of 95 °C for 5  s, 60 °C for 30 s; and a final extension step of 95 °C for 10 s, cooling to 65 °C for 60 s, and then heated to 97 °C for 1 s in the melting analysis. The gene-specific primers used β-actin as an internal control to normalize differences in template amounts, and the target genes contained C/EBPα, PPARγ, FABP4, SREBP-1c, ACC, FAS, ATGL, HSL, and AMPK (Supplementary Tables S1 and S2).

### 2.10. Protein Expression Assay

To analyze the protein expression, Western blot analysis (WB) was used to conduct this study. Cells were washed twice with PBS. Then, 70 μL of PI buffer was and scraped into a 1.7 mL tube, and the solution was homogenized by sonication (Qsonica, CT, USA) for 30 s. After the cells were homogenized, cell lysates were clarified by centrifugation at 15,000× *g* for 3 min at 4 °C. The protein concentration of the sample was determined by the Lowry method [21]. Then, the sample was analyzed by sodium dodecyl sulfate-polyacrylamide gel electrophoresis (SDS-PAGE), transferred to a polyvinylidene difluoride (PVDF) membrane, and subjected to WB analysis. Membranes were firstly probed with antibodies to phospho-AMPKα Thr172 (40H9; #2535), AMPKα (D5A2; #5831), and GAPDH (14C10; #2118) from Cell Signaling Technology (Danvers, MA, USA) and then probed with secondary rabbit antibodies (AB_2313567; Jackson ImmunoResearch Laboratories, West Grove, PA, USA) before the detection of the signal with an ECL substrate (Bio-Rad Laboratories, Hercules, CA, USA). Images were captured and quantified by using the BioRad Chemidoc and BioRad Image Lab software.

### 2.11. Statistical Assay

The statistical analysis was performed by using one-way ANOVA followed by Duncan’s and Tukey’s post hoc test to determine the significance of discrepancies between the two mean values. All statistical analyses were performed by using the statistical software package SPSS, version 20 (SPSS, Inc., Chicago, IL, USA). Statistically significant differences were identified where *p* < 0.05. The results of the cell experiment were presented from three independent experiments, and six independent experiments used in the animal experiment.

## 3. Results

### 3.1. Effect of LIM on Cell Viability and Proliferation Ability of 3T3-L1 Adipocytes

**Figure 1:**
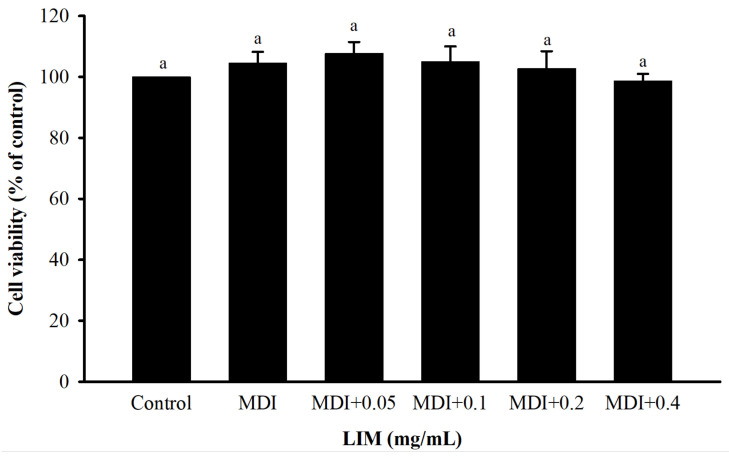
Effect of limonene (LIM) on cell viability in 3T3-L1 adipocyte. Preadipocytes were initiated to differentiate from the induced medium in the presence or absence of 0.05–0.4 mg/mL LIM. The histogram showed the relative fold change compared with the control group (set as 100%). The significant difference was evaluated by Tukey’s test for cell viability. Data were the means ± SD (*n* = 3). The same letter (a) means no statistical difference. The differentiation medium (MDI) (containing DMEM supplemented with IBMX (0.5 mM), dexamethasone (1 μM), and insulin (10 μg/mL)).

The effect of LIM treatments on 3T3-L1 adipocytes of cell viability and proliferation ability was assessed with an MTT assay (Figure 1). The result showed that the cell viability of 3T3-L1 adipocytes, treated with 0.05–0.4 mg/mL LIM, was not significantly different than the control group. This suggests that the LIM does not induce any cytotoxic effect on 3T3-L1 adipocytes up to a concentration of 0.4 mg/mL.

### 3.2. Effect of LIM on Lipid Accumulation in 3T3-L1 Adipocytes

**Figure 2:**
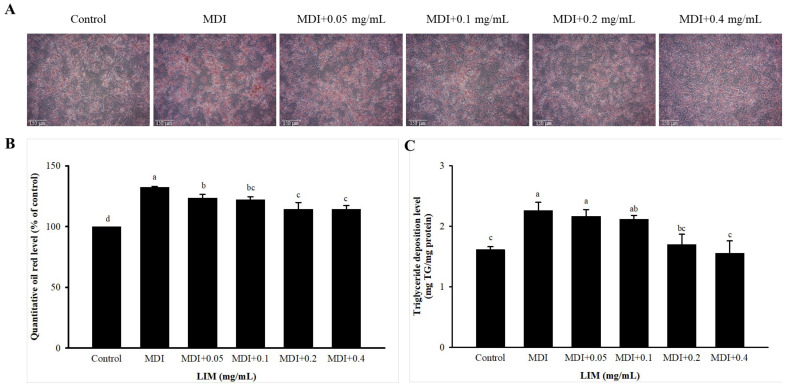
Effect of limonene (LIM) on lipid accumulation in 3T3-L1 adipocytes. (**A**) Intracellular lipid droplets were stained with oil red O and visualized by a microscope with 200× magnification. (**B**) The stained lipid droplets were solubilized with isopropanol and the absorbance was read at 510 nm using a microplate reader. (**C**) TG content assay of differentiated 3T3-L1 adipocytes treated with different concentrations of LIM. The cells were collected every two days, and the histogram showed the relative fold change compared with the control group (set as 100%). The significant difference was evaluated by Tukey’s test for lipid accumulation. Data were the means ± SD (*n* = 3). The values represented by different alphabets (a–c) were considered statistically significant between the different groups (*p* < 0.05). The differentiation medium (MDI) (containing DMEM supplemented with IBMX (0.5 mM), dexamethasone (1 μM), and insulin (10 μg/mL)).

The impact of LIM on the accumulation of intracellular lipid accumulation in 3T3-L1 adipocytes is depicted in Figure 2. The presence of lipid droplets was used as a marker of lipid accumulation. Microscopic observations of the oil red O staining revealed a reduction in the number of lipid droplets with increasing concentrations of LIM in a dose-dependent manner (Figure 2A). The oil red O content in 3T3-L1 adipocytes treated with MDI (MDI group) was significantly higher than that in the control group (Figure 2B). A range of 0.05–0.4 mg/mL LIM reduced oil red O content when compared with the MDI group (decreased by 6.7%, 7.8%, 13.7%, and 13.7%, respectively) (Figure 2B). Additionally, intracellular TG was quantified, and the results showed that 3T3-L1 adipocytes, treated with LIM at 0.2 and 0.4 mg/mL, decreased TG content by 24.7% and 31.1%, respectively, compared with the MDI group (Figure 2C). This suggests that LIM displays anti-adipogenic properties.

### 3.3. Effect of LIM on Protein and mRNA Expression Related to AMPK Signaling Pathway in 3T3-L1 Adipocytes

**Figure 3:**
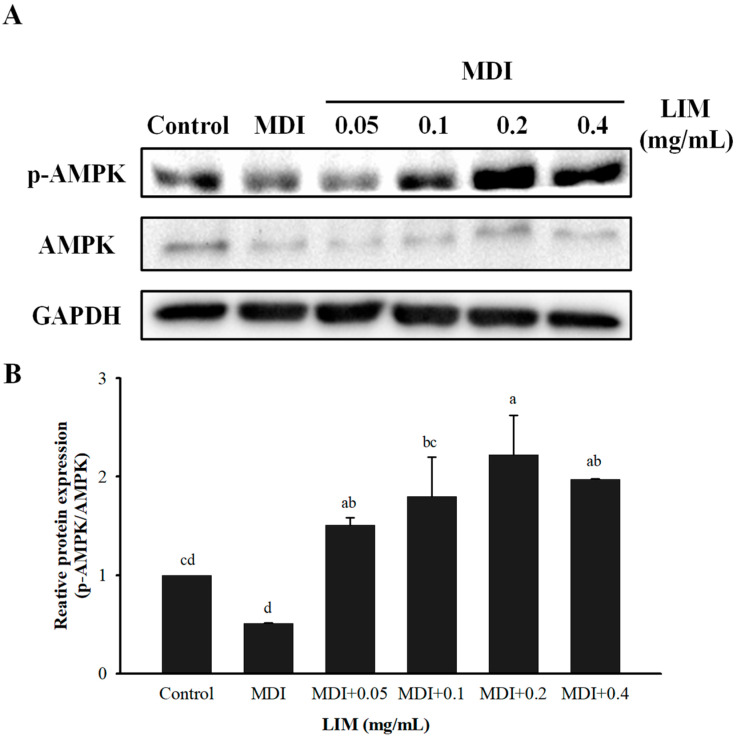
Effect of limonene (LIM) on protein expression of p-AMPK/AMPK in 3T3-L1 adipocytes. (**A**) Representative Western blot image showing the p-AMPK (62 kDa), AMPK (62 kDa), and GAPDH (37 kDa). (**B**) The graph shows the densitometric analysis of the p-AMPK/AMPK. The histogram showed the relative fold change compared with the control group. The significant difference was evaluated by Tukey’s test for lipid accumulation. Data were the means ± SD (*n* = 3). The values represented by different alphabets (a–d) were considered statistically significant between the different groups (*p* < 0.05). The differentiation medium (MDI) (containing DMEM supplemented with IBMX (0.5 mM), dexamethasone (1 μM), and insulin (10 μg/mL)); AMP-activated protein kinase (AMPK).

**Figure 4:**
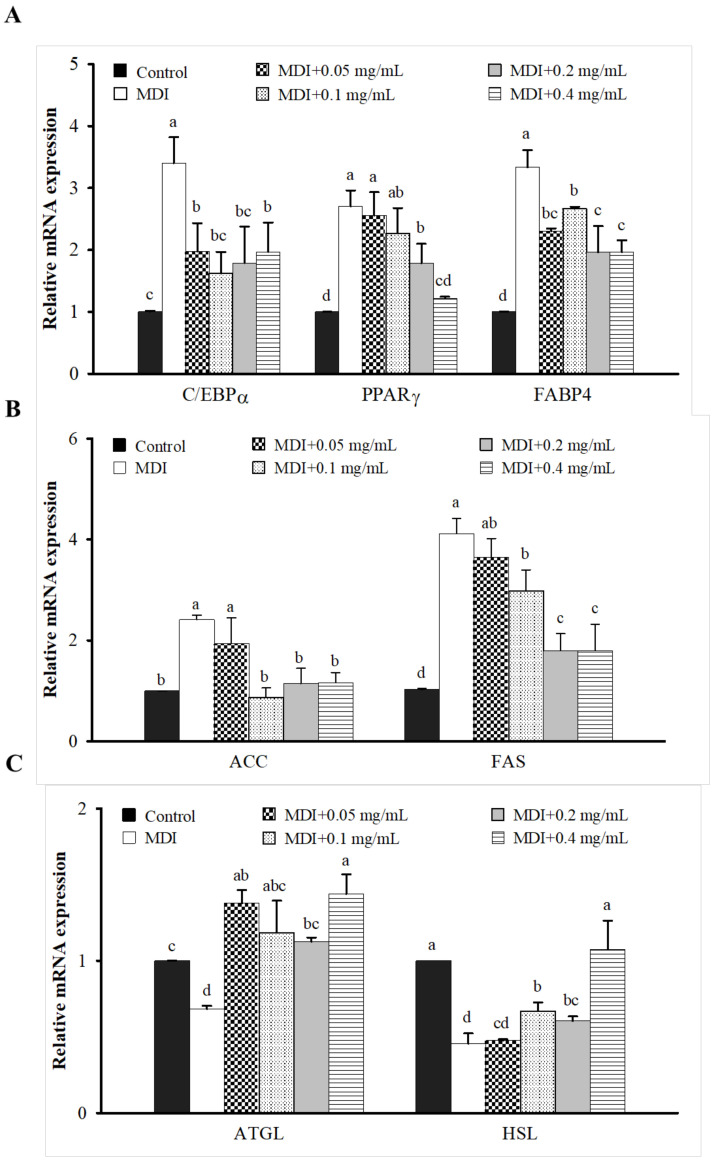
Effect of limonene (LIM) on lipid metabolism in 3T3-L1 adipocyte. The expression levels of mRNA are related to (**A**) adipogenesis, (**B**) lipogenesis and (**C**) lipolysis. The mRNA expressions of C/EBPα, PPARγ, FABP4, ACC, FAS, ATGL, and HSL were determined by qPCR. The histogram showed the relative fold change compared with the control group. The significant difference was evaluated by Tukey’s test for lipid accumulation. Data were the means ± SD (*n* = 3). The values, represented by different alphabets (a–d), were considered statistically significant between the different groups (*p* < 0.05). The differentiation medium (MDI) (containing DMEM supplemented with IBMX (0.5 mM), dexamethasone (1 μM), and insulin (10 μg/mL)); CCAAT/enhancer-binding proteins α (C/EBPα); peroxisome proliferator-activated receptor γ (PPARγ); fatty acid-binding protein 4 (FABP4); acetyl-CoA carboxylase (ACC); fatty acid synthase (FAS); hormone-sensitive lipase (HSL); adipose triglyceride lipase (ATGL).

The mechanism study of the AMP-activated protein kinase (AMPK) signaling pathway in 3T3-L1 adipocytes was performed via Western blot and qPCR methods to evaluate the expression levels of adipogenesis, lipogenesis, and lipolysis (Figure 3 and Figure 4). Compared with the MDI group, 0.05–0.4 mg/mL LIM significantly increased the protein expression of p-AMPK/AMPK (Figure 3A,B). The concentrations higher than 0.05 mg/mL LIM significantly decreased the mRNA expression of CCAAT/enhancer-binding proteins α (C/EBPα) and fatty acid-binding protein 4 (FABP4). Volumes of 0.2 and 0.4 mg/mL LIM significantly decreased the mRNA expression of peroxisome proliferator-activated receptor γ (PPARγ) (Figure 4A). Treatment with 0.1–0.4 mg/mL LIM significantly decreased the mRNA expression of acetyl-CoA carboxylase (ACC), and fatty acid synthase (FAS), and increased the mRNA expression of hormone-sensitive lipase (HSL) (Figure 4B,C). LIM of 0.05–0.4 mg/mL significantly increased the mRNA expression of adipose triglyceride lipase (ATGL) (Figure 4C). Conclusively, the study indicates that the lipid metabolism effect of LIM on 3T3-L1 adipocytes is mediated by the activation of AMPK.

### 3.4. Effect of LIM on the Growth Characteristics in High-Calorie Diet-Induced Obese Rats

The effect of the LIM on the growth characteristics in high-calorie diet-induced obese rats is shown in Table 1. After 16 weeks of high-calorie diet feeding, the body weight, weight gain, Lee’s index, and FCR levels of rats were significantly higher in the HD group than in the ND group. Body weight (reducing 10.1%, and 14.4%, respectively), weight gain and feed conversion ratio (FCR) levels were significantly lower in the LLIM and HLIM groups than in the HD group. Animals consuming a high-calorie diet saw non-significantly changes in food intake. The total fat tissue weight (reducing 29.94, and 36.5%, respectively) and Lee’s index were significantly lower in the LLIM and HLIM groups than in the HD group. The kidney weights of the HD and LLIM groups were significantly lower than in the ND group. LLIM and HLIM groups showed a non-significant difference in the liver, kidney, and spleen weights compared to the ND group.

### 3.5. Effect of LIM on Biochemical Parameters in High-Calorie Diet-Induced Obese Rats

Effect of LIM on biochemical parameters in high-calorie diet-induced obese rats (Table 2). Excessive fat accumulation often accompanies serum biochemicals metabolic disorder. The result showed that serum low-density lipoprotein cholesterol (LDLc), and uric acid levels of rats increased significantly higher in the HD group than in the ND group. The serum levels of triglyceride (TG) and LDLc were decreased in the HLIM group, compared with the HD group. HLIM group also increases the levels of high-density lipoprotein cholesterol (HDLc) and lipase levels. The levels of TC, free fatty acid (FFA), ketone body, aspartate aminotransferase (AST), alanine aminotransferase (ALT), sodium (Na), and potassium (K) for all groups were non-significant differences.

### 3.6. Effect of LIM on Epididymal Adipose Tissue Sections in High-Calorie Diet-Induced Obese Rats

**Figure 5:**
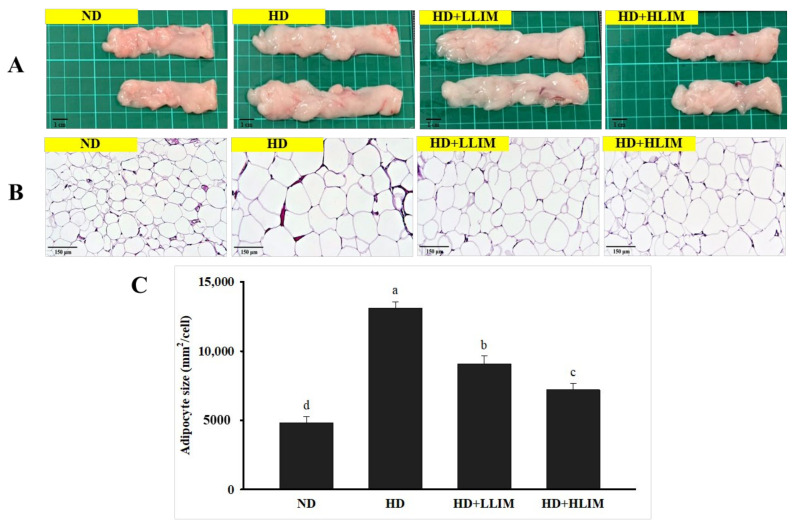
Effect of limonene (LIM) on morphological changes in epididymal adipose tissues of high-calorie diet-induced obese rats. (**A**) Histological observation. (**B**) H&E-stained epididymal adipose tissue sections. (**C**) Epididymal adipose size. Epididymal adipose tissue was stained with H&E and viewed under a microscope (×200). Data were the means ± SD (*n* = 6). The values represented by different alphabets (a–d) were considered statistically significant between the different groups (*p* < 0.05). The group names abbreviated are defined in Table 1 legend.

H&E staining was used to assess adipocyte size in epididymal adipose tissues. According to the H&E-stained epididymal adipose tissue section in high-calorie diet-induced rats, the high-calorie diet led to a pronounced expansion of adipocyte size compared with the ND group (Figure 5). The H&E-stained evaluation of epididymal adipose tissues in all LIM groups reduced the lipid accumulation; the volume of epididymal adipose tissues also became smaller and the size of epididymal adipocytes shrunk.

### 3.7. Effect of LIM on mRNA Expression of Genes Related to AMPK Signaling Pathway in High-Calorie Diet-Induced Obese Rats

**Figure 6:**
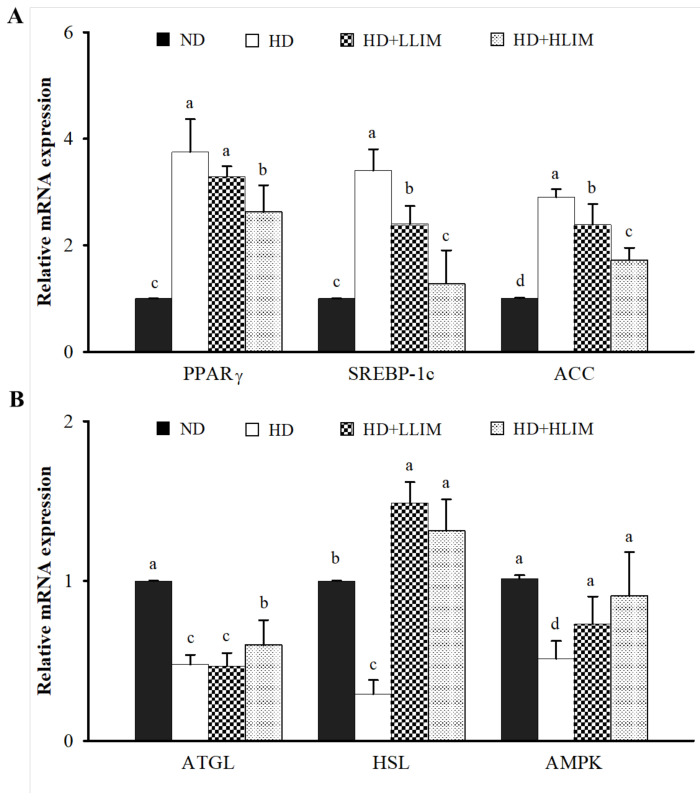
Effect of limonene (LIM) on mRNA expression of triglyceride metabolism in epididymal adipose tissue of high-calorie diet-induced obese rats. The expression levels of mRNA are related to (**A**) lipogenesis and (**B**) lipolysis. The mRNA expressions of PPARγ, ACC, ATGL, HSL, and AMPK were determined by qPCR. Data were the means ± SD (*n* = 6). The significant difference was evaluated by Duncan’s test for mRNA expression. Different lowercase letters (a–d) indicate a significant difference among groups (*p* < 0.05). The group names abbreviated are defined in Table 1 legend. Peroxisome proliferator-activated receptor γ (PPARγ); sterol regulatory element binding protein-1c (SREBP-1c); acetyl-CoA carboxylase (ACC); hormone-sensitive lipase (HSL); adipose triglyceride lipase (ATGL); AMP-activated protein kinase (AMPK).

The effect of LIM on mRNA expression related to AMPK signaling pathway in high-calorie diet-induced obese rats (Figure 6). When compared with the ND group, the HD group showed a significant increase in mRNA expression involved in adipogenesis (proliferator-activated receptor γ, PPARγ), and also in lipogenesis (sterol regulatory element binding protein-1c, SREBP-1c; acetyl-CoA carboxylase, ACC). Additionally, the HD group also showed a significant decrease in the mRNA expression involved in lipolysis (adipose triglyceride lipase, ATGL; hormone-sensitive lipase, HSL; AMP-activated protein kinase, AMPK). In contrast, lipogenesis-related mRNA expressions, such as SREBP-1c and ACC, were decreased. Conversely, lipolysis-related mRNA expression, such as HSL and AMPK in the epididymal adipose tissues, was increased by the LLIM group. The HLIM group also reduced adipogenesis-related mRNA expressions of PPARγ and promoted the mRNA expression of ATGL-related lipolysis.

## 4. Discussion

Obesity was a major risk factor for chronic diseases such as coronary heart disease, hypertension, type 2 diabetes, and some types of cancer [3]. Encouragingly, more and more evidence of LIM has shown the effect of LIM on anti-obesity [18,20]. Several recent studies reported the best target is to inhibit the fat mass and obesity-associated protein (FTO) in obesity therapy [25]. Studies on animal models revealed that homeostasis energy and metabolic disturbances in obesity were associated with the functionality of FTO [26]. Previous studies have found that LIM has low binding energy to FTO, the most relevant handle point in obesity treatment. As such, LIM was found to be a good, competitive inhibitor to block adipogenesis-related transcription factors [27].

Moreover, the AMPK signaling pathway was an important target for the treatment of obesity [28]. Previous studies have shown that the adipogenesis (PPARγ, C/EBPα, FABP4), and lipogenesis (SREBP-1c, ACC, FAS) were inhibited and the lipolysis (ATGL, HSL) was promoted by phosphorylating AMPK [8,9,29].

3T3-L1 adipocytes are the most commonly used of adipocyte cell lines in the study of lipid metabolism [30]. Understanding the effect of lipid metabolism has been considered a key issue for the development of anti-obesity agents [31]. Jing et al. (2013) showed that 0.0068 mg/mL LIM blocked lipid accumulation in 3T3-L1 adipocytes [19]. Tan et al. (2016) found that 3T3-L1 adipocytes treatment with 0.00014 mg/mL LIM increased the free glycerol in the medium [32]. Ngamdokmai et al. (2021) showed that 0.05 and 0.1 mg/mL LIM reduced oil red O levels in 3T3-L1 adipocytes [33]. Soundharrajan et al. (2018) found that LIM at 0.00014 mg/mL significantly downregulated the mRNA expressions of PPARγ, C/EBP-α, C/EBP-β, SREBP-1, FAS, and adiponectin in differentiated adipocytes [34]. These results suggest that LIM is effective at inhibiting adipocyte lipid accumulation, but few studies have reported on the effect of LIM on anti-obesity-related metabolism. In this study, the results indicated that a range between 0.2 and 0.4 mg/mL LIM mitigated the lipid accumulation in 3T3-L1 adipocytes. LIM increased the phosphorylation of AMPK, while decreasing the mRNA expression of C/EBPα, PPARγ, FABP4, ACC, and FAS in 3T3-L1 adipocytes. The phosphorylation of AMPK also increased the mRNA expression of ATGL and HSL in 3T3-L1 adipocytes. Therefore, the anti-adipogenic influence of AMPK is recognized as a major effect of LIM in 3T3-L1 adipocytes.

Obesity is generally associated with lipid accumulation of adipose tissues and dyslipidemia. This study’s results indicate that LIM reduced the body weight, total fat tissue weight, adipose cell size, serum TG, and LDL. At the same time, LIM increased the content of serum HDL in HD treatment rats. Khan et al. (2019) found that LIM (250 mg/kg) improved the dyslipidemia of high-fat diet-induced rats by decreasing serum TG, and LDL and increasing HDL levels [35]. In addition, previous studies have found that the normal values of serum LDLc and HDLc in rats are 15.58–35.09 and 36.78–54.65, respectively [36]. In this study, the serum LDLc and HDLc ranges are 9.50–12.6 and 41.67–51.0. It is believed that, although there are few differences, they are not significant. This study showed that LIM reduced the lipid anabolism of high-calorie diet-induced obese rats by decreasing the mRNA expression of PPARγ, SREBP-1c, and ACC. Additionally, LIM promoted the lipid catabolism of high-calorie diet-induced obese rats by increasing the mRNA expression of ATGL, HSL, and AMPK. The result of mRNA expression in epididymal adipose tissues may explain the mechanism of lipid metabolism in high-calorie diet-induced obese rats.

Previous studies showed that daily administration of LIM, at a dose below 1650 mg/kg, has no toxicity in rats [15]. Therefore, the animal dose (below 1000 mg/kg) of LIM was safe. In this study, the animal dose of LLIM (154 mg/kg) was selected from this cell model study by calculating the blood volume and body weight of rats, and HLIM (1000 mg/kg) was based on the previous study by converting the food intake of the rats [18]. Santiago et al. (2010) showed that administration of LIM (added 2% LIM in a high-fat diet) for 4 weeks reduced body weight in high-fat diet-induced rats [18]. However, the previous study had a number of research limits. For example, LIM was added to the high-fat diet, and so it could not calculate the dose accurately. Additionally, only using one dose of LIM, we could not establish a dose below 1000 mg/kg for anti-obesity effects. In this study, two doses (154 and 1000 mg/kg) were fed via oral gavage to calculate the LIM intake. In this study, both LLIM (154 mg/kg) and HLIM (1000 mg/kg) were seen to have anti-obesity properties. However, an ideal drug candidate would have high potency, and ideally at low doses to achieve adequate efficacy [37]. Therefore, we consider LLIM (154 mg/kg) to be the optimal dose for anti-obesity.

Previous studies have shown that, although citrus peel is a waste material, it contains a variety of bioactive components. Huang et al. (2022) found that the amounts of limonene, nobiletin, and 3-methoxynobiletin in citrus peel were markedly increased after fermenting citrus peels [38]. Therefore, our research team is conducting experiments to extract limonene from lemon peel. Additionally, we will conduct further evaluations of the dose and efficacy of limonene, present in lemon peel, in order to achieve the effect of a circular economy.

## 5. Conclusions

The use of LIM can reduce obesity by activating the AMPK signaling pathway, and 154 mg/kg LIM can be an optimally effective dose for anti-obesity effects. Collectively, our findings provide novel insights into the physiological roles of LIM in the regulation of metabolism in obesity.

## Figures and Tables

**Table 1:** Effect of limonene (LIM) on the growth characteristics in high-calorie diet-induced obese rats.

	ND			HD			HD + LLIM			HD + HLIM		
Body weight (g)	493.50	±	9.32 ^b^	564.25	±	47.14 ^a^	503.13	±	42.60 ^b^	477.23	±	41.07 ^b^
Weight gain (%)	94.15	±	4.39 ^c^	123.38	±	16.38 ^a^	111.01	±	3.63 ^b^	100.37	±	6.52 ^bc^
Lee’s index (g/cm)	0.30	±	0.01 ^c^	0.35	±	0.01 ^a^	0.32	±	0.00 ^b^	0.30	±	0.00 ^c^
Food intake (g/rat/day)	25.89	±	1.07 ^a^	18.23	±	0.96 ^b^	19.02	±	1.11 ^b^	19.11	±	0.87 ^b^
Feed conversion ratio (%)	8.77	±	0.37 ^d^	16.12	±	1.21 ^a^	13.41	±	0.36 ^b^	12.04	±	0.57 ^c^
Liver (%)	3.46	±	0.30 ^a^	3.22	±	0.27 ^a^	3.50	±	0.41 ^a^	3.63	±	0.44 ^a^
Kidney (%)	0.81	±	0.06 ^a^	0.65	±	0.04 ^b^	0.69	±	0.11 ^b^	0.82	±	0.05 ^a^
Spleen (%)	0.19	±	0.01 ^a^	0.17	±	0.02 ^a^	0.17	±	0.01 ^a^	0.16	±	0.02 ^a^
Epididymal adipose tissue (%)	1.55	±	0.18 ^b^	2.83	±	0.94 ^a^	2.22	±	0.46 ^ab^	1.83	±	0.35 ^b^
Mesenteric adipose tissue (%)	1.02	±	0.04 ^c^	1.90	±	0.15 ^a^	1.43	±	0.20 ^b^	1.17	±	0.16 ^c^
Retroperitoneal adipose tissue (%)	1.23	±	0.05 ^c^	3.16	±	0.64 ^a^	2.04	±	0.43 ^b^	2.05	±	0.17 ^b^
Total fat tissue (%)	3.81	±	0.59 ^c^	8.25	±	1.11 ^a^	5.78	±	1.02 ^b^	5.24	±	0.58 ^b^

Data were the means ± SD (*n* = 6). The significant difference was evaluated by Duncan’s test for growth characteristics. The values represented by different alphabets (a–c) were considered statistically significant between the different groups (*p* < 0.05). Normal diet (ND); high-calorie diet (HD); HD with low-dose LIM (HD + LLIM); HD with high-dose LIM (HD + HLIM); feed conversion ratio (FCR).

**Table 2:** Effect of limonene (LIM) on biochemical parameters in high-calorie diet-induced obese rats.

	ND			HD			HD + LLIM			HD + HLIM		
Triglyceride (mg/dL)	75.11	±	7.26 ^a^	84.05	±	4.54 ^a^	74.10	±	9.51 ^a^	45.79	±	1.81 ^b^
Total cholesterol (mg/dL)	78.84	±	2.71 ^a^	72.36	±	2.16 ^b^	77.95	±	2.78 ^a^	73.29	±	2.84 ^b^
Low-density lipoprotein cholesterol (mg/dL)	9.50	±	0.58 ^c^	12.67	±	0.58 ^a^	11.00	±	0.00 ^b^	11.67	±	0.58 ^b^
High-density lipoprotein cholesterol (mg/dL)	48.00	±	5.48 ^ab^	41.67	±	2.31 ^b^	43.67	±	2.08 ^ab^	51.00	±	6.00 ^a^
Free fatty acid (mmol/L)	1.44	±	0.12 ^a^	1.32	±	0.23 ^a^	1.43	±	0.06 ^a^	1.38	±	0.13 ^a^
Lipase (mmol/L)	7.20	±	0.45 ^a^	6.40	±	0.55 ^b^	6.57	±	0.53 ^ab^	7.20	±	0.45 ^a^
Ketone body (mmol/L)	1.24	±	0.21 ^a^	1.52	±	0.13 ^a^	1.34	±	0.15 ^a^	1.42	±	0.16 ^a^
Aspartate aminotransferase (U/L)	136.20	±	28.60 ^a^	134.60	±	7.64 ^a^	131.29	±	12.40 ^a^	135.35	±	27.32 ^a^
Alanine aminotransferase (U/L)	50.20	±	5.26 ^a^	49.40	±	3.83 ^a^	45.79	±	7.38 ^a^	43.67	±	5.13 ^a^
Creatinine (mg/dL)	0.71	±	0.03 ^a^	0.75	±	0.08 ^a^	0.67	±	0.06 ^ab^	0.62	±	0.06 ^b^
Uric acid (mg/dL)	2.18	±	0.33 ^b^	3.10	±	0.56 ^a^	3.92	±	0.59 ^a^	3.13	±	0.57 ^a^
Sodium (mmol/L)	150.82	±	2.50 ^a^	150.33	±	2.52 ^a^	150.69	±	2.20 ^a^	149.65	±	2.56 ^a^
Potassium (mmol/L)	7.22	±	1.61 ^a^	7.16	±	0.67 ^a^	7.99	±	0.79 ^a^	7.78	±	0.80 ^a^

Data were the means ± SD (*n* = 6). The significant difference was evaluated by Duncan’s test for biochemical parameters. The values represented by different alphabets (a–c) were considered statistically significant between the different groups (*p* < 0.05). The abbreviated group names are defined in the Table 1 legend.

## Data Availability

Data are available from the corresponding author upon reasonable request.

## Supplementary Materials

The following supporting information can be downloaded at https://www.mdpi.com/article/10.3390/nu15020267/s1, Table S1: Specific primer of qPCR analysis in 3T3-L1 adipocytes, Table S2: Specific primer of qPCR analysis in high-calorie diet-induced obese rats.
