# Supplementary material for: D-Limonene Promotes Anti-Obesity in 3T3-L1 Adipocytes and High-Calorie Diet-Induced Obese Rats by Activating the AMPK Signaling Pathway
Source: Nutrients. 2023 Jan 4;15(2):267. doi: 10.3390/nu15020267 (PMC9861755; doi:10.3390/nu15020267)
Supplement: Supplementary file 1 [file nutrients-15-00267-s001.zip › Supplementary Table S1.pdf]

Supplementary Table S1. Specific primer of qPCR analysis in 3T3-L1 adipocytes.

Gene	Primer sequence (5′-3′)	Reference (NCBI GenBank)
β-actin	F-TCTGGCACCACACCTTCTACAA<br>R-TTTTCACGGTTGGCCTTAGG	NM007393.5
ACC	F-TTGCAGAAGAAATACGCCATATGT<br>R-AGGGCACTGACTCTCTTATAATCCT	NM133360.2
ATGL	F-ACTCACATCTACGGAGCCTCG<br>R-TCCTTGGACACCTCAATAATGTTG	NM001163689.1
C/EBPα	F-CTGGAGTTGACCAGTGACAATGA<br>R-AGTTGCCCATGGCCTTGAC	NM001287514.1
FABP4	F-GAGGCGGATGAGAACAAGCA<br>R-CTCCCAGCAGCTACCATGGA	NM024406.3
FAS	F-CACAATGGACCCCCAGCTT<br>R-AGACGCCAGGTTCGTTCCT	NM007988.3
HSL	F-CTCATGGCTCAACTCCTTCCTG<br>R-TGCCTCAGACACACTCCTGC	NM010719.5
PPARγ	F-TCTCCTGTTGACCCAGAGCAT<br>R-TGGGCCAGAATGGCATCT	NM001174097.2
