# Supplementary material for: D-Limonene Promotes Anti-Obesity in 3T3-L1 Adipocytes and High-Calorie Diet-Induced Obese Rats by Activating the AMPK Signaling Pathway
Source: Nutrients. 2023 Jan 4;15(2):267. doi: 10.3390/nu15020267 (PMC9861755; doi:10.3390/nu15020267)
Supplement: Supplementary file 1 [file nutrients-15-00267-s001.zip › Supplementary Table S2.pdf]

Supplementary Table S2. Specific primer of qPCR analysis in high-calorie diet-induced obese rats.

Gene	Primer sequence (5′-3′)	Reference (NCBI GenBank)
β-actin	F-AGGCCCCTCTGAACCCTAAG<br>R-CAGCCTGGATGGCTACGTACA	NM031144.3
ACC	F- GCTGGGACAAAGAACCATCC<br>R- TCCGTTGTTGTGCATTATCTGG	NM022193.1
AMPK	F-GTGGATCGCCAAATTATGCA<br>R-AACCTCAGGACCCGCATACA	NM023991.1
ATGL	F-GGCCACTGCCATGATGGTA<br>R-GCAGCCACTCCAACAAACG	NM001108509.2
FAS	F-TCTCCTGTTGACCCAGAGCAT<br>R-TGGGCCAGAATGGCATCT	NM017332.1
HSL	F-GTCACGCTACATAAAGGCTGCTT<br>R-CAGCCCGATGGAGAGAGTCT	NM012598.1
PPARγ	F-AAGTTTGAGTTTGCTGTGAAGTTCA<br>R-CGATGGGCTTCACGTTCAG	NM001145366.1
SREBP-1c	F-CGGGACAGCTTAGCCTCTACA<br>R-CGGCCACAAGAAGTAGATCA	NM001276707.1
